# Whole genome amplification of cell-free DNA enables detection of circulating tumor DNA mutations from fingerstick capillary blood

**DOI:** 10.1038/s41598-018-35470-9

**Published:** 2018-11-23

**Authors:** Rekha Gyanchandani, Erik Kvam, Ryan Heller, Erin Finehout, Nicholas Smith, Karthik Kota, John R. Nelson, Weston Griffin, Shannon Puhalla, Adam M. Brufsky, Nancy E. Davidson, Adrian V. Lee

**Affiliations:** 1 Women’s Cancer Research Center, Department of Pharmacology and Chemical Biology, UPMC Hillman Cancer Center, Magee Womens Research Institute, Pittsburgh, PA 15213 USA; 2 GE Global Research, One Research Circle, Niskayuna, NY 12309 USA; 3 Department of Medicine, University of Pittsburgh, Pittsburgh, PA 15213 USA; 4 Present Address: Western Oncolytics, 265 William Pitt Way, Pittsburgh, PA 15238 USA; 5 Present Address: QIAGEN, 100 Cummings Center, Beverly, MA 01915 USA; 6 Present Address: Terumo BCT, 10811 Collins Ave, Lakewood, CO 80215 USA; 7 Fred Hutchinson Cancer Research Center, 1100 Fairview Avenue N, Seattle, WA 98109 USA

## Abstract

The ability to measure mutations in plasma cell-free DNA (cfDNA) has the potential to revolutionize cancer surveillance and treatment by enabling longitudinal monitoring not possible with solid tumor biopsies. However, obtaining sufficient quantities of cfDNA remains a challenge for assay development and clinical translation; consequently, large volumes of venous blood are typically required. Here, we test proof-of-concept for using smaller volumes via fingerstick collection. Matched venous and fingerstick blood were obtained from seven patients with metastatic breast cancer. Fingerstick blood was separated at point-of-care using a novel paper-based concept to isolate plasma centrifuge-free. Patient cfDNA was then analyzed with or without a new method for whole genome amplification via rolling-circle amplification (WG-RCA). We identified somatic mutations by targeted sequencing and compared the concordance of mutation detection from venous and amplified capillary samples by droplet-digital PCR. Patient mutations were detected with 100% concordance after WG-RCA, although in some samples, allele frequencies showed greater variation likely due to differential amplification or primer inaccessibility. These pilot findings provide physiological evidence that circulating tumor DNA is accessible by fingerstick and sustains presence/absence of mutation detection after whole-genome amplification. Further refinement may enable simpler and less-invasive methods for longitudinal or theranostic surveillance of metastatic cancer.

## Introduction

The recent revolution in massively parallel DNA sequencing is unravelling the genomic basis of primary cancer^1^ and providing important examples of genomic evolution and the hierarchal order of cancer mutations^2^, convergent evolution^3^, and the development of drug resistance^4^. However, there are limited studies of genomic changes in lethal metastatic disease, mainly due to challenges in obtaining biopsies on advanced cancers. The transformative findings that somatic DNA mutations are present within plasma cell-free DNA (cfDNA) led to the notion of ‘liquid biopsy’, an attractive and less-invasive alternative to solid tumor biopsy for the discovery and longitudinal monitoring of somatic mutations^5–9^. Despite immense potential, liquid biopsies are not yet widely implemented in the clinic^10^. The natural low abundance of cfDNA—and/or circulating tumor DNA (ctDNA) comprised therein—in blood and other bodily fluids remains a significant challenge for assay development and clinical translation of liquid biopsies, especially combined with other key contributing factors, such as tumor biomarker availability, minimum-analysis testing requirements, and pre-analytical collection variables^10,11^. Current methods for liquid biopsies require at least 10 mL of blood by venipuncture, and larger volumes may need to be collected to guarantee a sufficient, but precious, supply of cfDNA for clinical study^6,12^. Studies have shown wide and unpredictable ranges of cfDNA in blood from cancer patients^13,14^. Therefore, for some patients, yields of cfDNA from milliliters of blood may not be sufficient to support workflows that require high amounts of template, such as DNA sequencing and PCR with technical replicates. These volumetric and concentration demands often present a challenge to existing liquid biopsy paradigms.

DNA pre-amplification methods are widely used to meet minimum-input requirements for downstream molecular analysis. We have previously shown that targeted pre-amplification of a stretch of cfDNA containing a mutation enables generation of sufficient template amounts for droplet digital PCR (ddPCR), while preserving the linearity of mutant allele detection^15^. However, this targeted PCR-based approach is low throughput and multiplexable for only a few known mutations. Whole-genome amplification (WGA) methods have been developed for single-cell analyses such as sequencing circulating tumor cells in breast cancer^16^, and applied to cfDNA, however, few if any WGA methods have been truly optimized for naturally short and fragmented cfDNA templates, especially when cfDNA is present in only finite or trace amounts. For example, cfDNA is a poor template for multiple displacement amplification (MDA)^17^, even though MDA is proficient for single-cell analysis of single genomes (~6.6 pg) comprising long and intact genomic DNA. Furthermore, to achieve optimal results, many WGA kits recommend using a higher template input amount (e.g. 5–10 ng) than what is often isolated from 10 mL of blood for some patients. Thus, better methods are required in the liquid biopsy community for representative amplification of small amounts of cell-free DNA for robust characterization and detection of ctDNA.

Liquid biopsies are also challenged by pre-analytical collection variables that are introduced during blood handling and shipping^11^. Specifically, the contamination of cfDNA with free genomic DNA (gDNA) released by resident blood cells is a documented problem in blood collection tubes that experience even small delays in processing. *Ex vivo* gDNA contamination occurs through a time-, temperature-, and agitation-dependent manner and increases signal background, thereby reducing sensitivity when analyzing cfDNA^11,18,19^. Consequently, blood must be centrifuged as soon as possible to collect and freeze plasma specimens for archival analysis. This process is not always operationally feasible and adds complexity to multicenter trials where samples may be transported or handled slightly differently. While Cell-Free DNA BCT^TM^ tubes from Streck have been developed to preserve cfDNA integrity and delay centrifugation for up to several days^20^, temperature fluctuations can occur during sample handling and lead to genomic contamination events^21,22^. Thus, inexpensive and robust methods for collecting plasma and preserving cfDNA are still unmet needs.

Herein, we utilize a novel paper-based device concept (termed “PlasmaClip”) for centrifuge-free isolation of plasma from fingerstick blood (~75 μL) at the point-of-care, and a new method for whole genome amplification of cfDNA via rolling-circle amplification (WG-RCA), to evaluate the feasibility for fingerprick-based detection of circulating tumor DNA. This study provides a clinical proof-of-concept for fingerstick-detection of somatic variants from advanced cancer patients, which upon further development such as improvement in allele frequency quantification may enable simpler and less-invasive methods for longitudinal or theranostic surveillance of metastatic cancer.

## Results

### Study design

**Figure 1:**
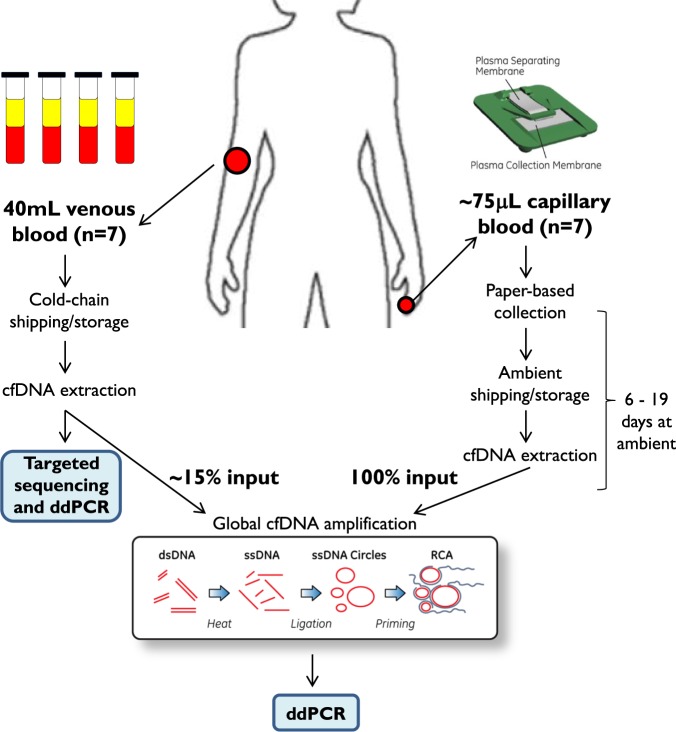
Study design. Venous whole blood was drawn in Streck cell-free DNA blood tubes from seven patients with metastatic breast cancer. Concurrent fingerstick capillary blood samples were collected from the same individuals onto a novel paper-based PlasmaClip device. Cell-free DNA (cfDNA) was isolated from venous and fingerstick blood samples and then subjected to a new method for whole genome amplification via rolling-circle amplification (WG-RCA). Targeted sequencing was used to pre-identify somatic mutations from venous unamplified cfDNA, and concordance testing was conducted in WG-RCA samples from venous and fingerstick cfDNA by droplet digital PCR (ddPCR).

Conventional liquid biopsy methods require up-front collection and processing of large volumes of venous blood (≥10 mL) to obtain sufficient quantities of cfDNA. Here, a novel whole genome amplification method (WG-RCA) was tested to representatively amplify low quantities of cfDNA via rolling-circle amplification (Fig. 1, inset). We used targeted sequencing to pre-identify somatic mutations by conventional liquid biopsy of venous blood and then compared the concordance rate of mutation detection with and without WG-RCA by highly sensitive droplet digital PCR (ddPCR). Simultaneously, fingerstick blood (~75 μl) was collected onto a paper-based device assembly (PlasmaClip) for centrifuge-free isolation of plasma at the point-of-care. Following PlasmaClip-based sample extraction and WG-RCA, the concordance of somatic mutation detection was evaluated by ddPCR between fingerstick and venous cfDNA samples. A schematic overview of our study design is provided in Fig. 1, and each step is described in detail below with supporting results.

### Patient sample collection and baseline sequencing results for 79 genes frequently mutated in breast cancer

By standard liquid biopsy, venous whole blood was collected from 7 patients with ER-positive metastatic breast cancer into Streck Cell-Free DNA blood tubes to isolate genomic DNA from buffy coat and cfDNA from plasma. Clinical histories for each patient are provided in Supplementary Table S1. Patient DNA was separately purified from cell-free plasma and peripheral blood mononuclear cells (buffy coat) using standard techniques as described in the methods section. Targeted sequencing was performed using the MammaSeq^TM^ breast cancer panel^23^ to pre-identify somatic variants in cfDNA that were absent in patient-matched germline DNA. MammaSeq is a breast cancer specific NGS panel, targeting 79 genes and 1369 mutations, optimized for use in primary and metastatic breast cancer. MammaSeq was validated on 46 solid tumors where it showed 100% concordance with Ampliseq™ Cancer Hotpot Panel v2 previously run on these samples in a CLIA laboratory^23^. We have also conducted MammaSeq on venous cfDNA and performed orthogonal validation with ddPCR^23^. In this manuscript we leveraged MammaSeq data on venous plasma samples from patients CF20, CF22, CF23, and metastatic tumor samples from CF4, CF23, and CF31 described in our previous study^23^. Hence, for 2 patients in this study (CF4 and CF23), MammaSeq data on tumors could be compared against fresh blood samples, albeit at non-concurrent samplings (i.e. blood samples were collected 7 and 12 months after metastatic tumor biopsy, respectively). In contrast, for another patient (CF31), blood was obtained ~1.5 months *prior* to the results of metastatic tumor biopsy but an insufficient plasma volume was banked that yielded less than the required ~20 ng of input cfDNA for MammaSeq analysis. Hence, it could not be sequenced. This technical limitation exemplifies one of our motivations for investigating efficient whole-genome amplification techniques for plasma cfDNA.

As shown in Supplementary Table S2, circulating mutations were identified in 3 out of the 6 patients (50%) who had sufficient venous cfDNA for MammaSeq analysis. For two patients with non-concurrent blood and tumor specimens, one patient (CF4) exhibited a tumor-matched variant in active circulation, while the other (CF23) showed no matching tumor variant in circulation (Supplementary Table S2). Overall, 5 total patients presented with somatic variants in *PIK3CA* (incidence of 5/7, 71%) in at least tumor or blood. Two patients additionally presented with somatic variants in either *ESR1* or *KRAS* (incidence of 1/7, 14%, respectively). These findings are consistent with other breast cancer studies showing that *PIK3CA* lesions are the most frequent mutation in ER-positive cancer^24^, while mutations in *ESR1* and *KRAS* may emerge in advanced disease as a response to drug intervention^15,25,26^.

### Centrifuge-free isolation of plasma from fingerstick blood using PlasmaClip

**Figure 2:**
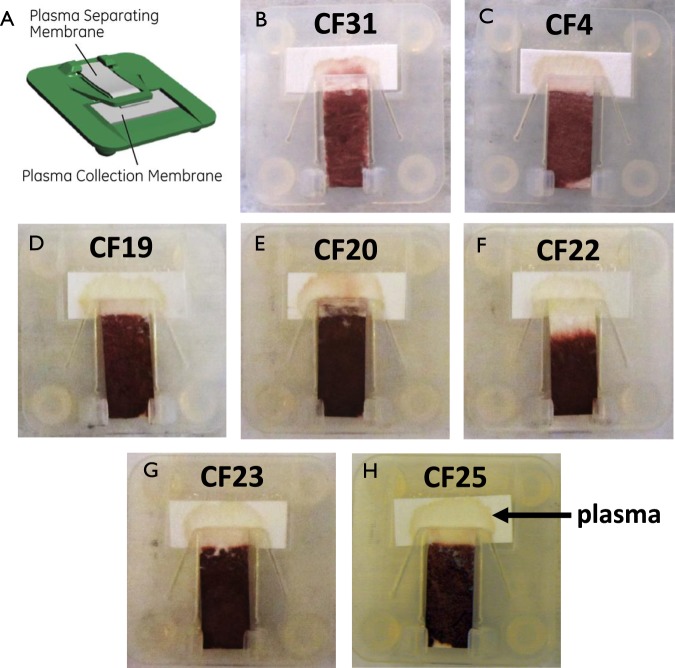
Fingerstick blood collection using paper-based PlasmaClip device. The PlasmaClip device consists of a commercial plasma separation membrane and a collection pad held together in a reversible manner such that the entire dried plasma spot can be easily removed and processed for cfDNA extraction. Fingerstick blood was applied onto the plasma separating membrane and the resulting plasma sample is indicated for the cohort of 7 patients with metastatic breast cancer.

Simultaneous to venous blood collection, we collected patient-matched fingerstick samples onto a novel paper-based device termed PlasmaClip. PlasmaClip utilizes a commercial glass fiber material for rapidly separating plasma at the point-of-collection^27^ and stores fingerstick cell-free DNA in a dry, ambient state on commercial cellulosic filter paper (Fig. 2). The PlasmaClip device positions the plasma separation and collection membranes together in a reversible manner (similar to a paper clip) such that an entire dried plasma spot can be easily removed and processed for cfDNA extraction (Fig. 2, inset A). At GE Global Research, a fingerstick procedure using standard lancing equipment was first investigated on healthy donors to develop a methodology for collecting samples without genomic DNA contamination from interstitial fluids (see Supplementary Fig. S1). Fingerprick samples were then obtained from 7 patients with metastatic breast cancer and deposited onto PlasmaClip devices as shown in Fig. 2. The hospital collection method at UPMC was adjusted after patient CF31 to ensure that alcohol-wiped fingertips were fully-dried prior to lancing, which diminished visual evidence of spurious hemolysis on the plasma collection pad among the remaining collections (Fig. 2, panels C–H) as we also observed in our pre-optimization studies (Supplementary Fig. S1). A single dried plasma spot was obtained from each breast cancer patient and stored at room temperature for a total of 6–19 days (see Supplementary Table S3) prior to cfDNA extraction.

### Whole genome amplification to expand the available pool of cfDNA for molecular analysis

Low amounts of cfDNA isolated from venous plasma is a technical challenge for somatic mutation detection, and very low fingerstick volumes of blood further compound this critical issue. We hypothesized that whole genome amplification (WGA) of cfDNA could increase cfDNA supply from both venous and fingerstick cfDNA by representatively expanding the pool of cfDNA available for molecular analysis, and therefore obviate conventional practices for over-collection. A proprietary WGA methodology comprising rolling-circle amplification (WG-RCA) was pre-developed with healthy-donor venous plasma to achieve global and representative amplification of cfDNA (Supplementary Fig. S2). Briefly, cfDNA was heated to individual strands, ligated to form single-stranded DNA circles, and amplified using phi29 polymerase and exonuclease-resistant random primers by an exponential rolling circle mode of amplification^28^ (Fig. 1). All steps are performed in a single-tube format to avoid intermediate sample clean-up and therefore minimizes physical template loss during processing. Using targeted sequencing of 409 genes (Ion AmpliSeq^TM^ Comprehensive Panel) in healthy-donor cfDNA, WG-RCA showed strikingly higher average depth of gene coverage and reduced allelic imbalance compared to conventional multiple strand-displacement amplification (MDA) when using a limiting amount of cfDNA template (~1 ng) (Supplementary Fig. S2). These findings agree with prior studies that standard MDA methods do not efficiently amplify fragmented DNA below 1 kB and therefore are functionally mismatched with naturally-fragmented cfDNA templates due to high allelic dropout and amplification failures^17^. WG-RCA solves this issue by ligating cfDNA into single-stranded circles that are better templates for strand-displacement amplification, similar to published cfDNA amplification methods involving double-stranded DNA circular templates^17^.

### Comparison of patient cfDNA yields before and after WG-RCA

**Figure 3:**
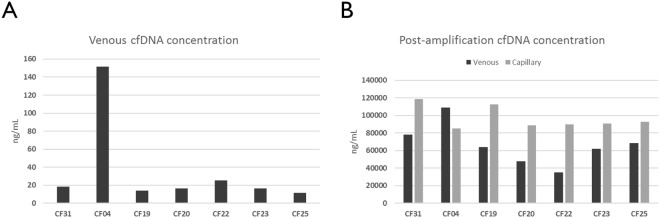
Comparison of patient cfDNA yields before and after WG-RCA. (**A**) Among 7 patients with metastatic breast cancer, baseline venous plasma cfDNA yields ranged between 11–152 ng per mL. (**B**) Using ~15% of venous cfDNA and 100% of fingerpick cfDNA as input for whole genome rolling-circle amplification (WG-RCA) resulted in greater than 1000-fold amplification of DNA for downstream applications.

Among seven patients with metastatic breast cancer, venous plasma yielded 11–152 ng of cfDNA per mL, which is consistent with ranges previously reported^29^. Six of these patients possessed less than 30 ng/mL (Fig. 3A), highlighting that the amount of cfDNA from a patient is unpredictable, and in some cases, could be insufficient to support initial or confirmatory molecular analyses as others have reported^30^. However, amplifying a small amount (~15%) of the purified venous cfDNA pool as template for WG-RCA achieved greater than 1,000-fold cfDNA amplification and generated micrograms of DNA for testing and analysis (Fig. 3B). Slightly higher amplification yields were observed from PlasmaClip-based samples (Fig. 3B), in which ~100% of the purified fingerstick cfDNA pool was used as template. However, different cfDNA purification methods were used to extract venous and fingerstick plasma samples (i.e. silica membrane-based kit and precipitation-based kit, respectively), which may explain the differences observed among our WG-RCA amplification yields. Due to limiting sample volume, we were not able to measure the starting concentration of cfDNA purified from fingerstick plasma, but other studies have demonstrated capillary cfDNA concentrations to be ~50% lower than venous levels on average (i.e. approximately 10 ng/mL or 10 pg/μL)^31,32^. However, there is growing evidence that substantial venous cfDNA losses occur during silica membrane-based purification (unlike precipitation-based methods)^33–35^, which may account for the observed trend in WG-RCA yields (Fig. 3B). Nevertheless, large amounts of amplified cfDNA were generated from all venous and fingerstick samples using WG-RCA, thereby enabling deep replicate studies for each patient.

We sequenced patient CF4 cfDNA using the Ion AmpliSeq^TM^ Comprehensive Cancer Panel and confirmed that WG-RCA of venous cfDNA improves gene coverage and decreases allelic imbalance relative to an MDA kit (REPLI-g Mini Kit, Qiagen) despite using an excess input of cfDNA (~35–40 ng) for whole genome amplification (Supplementary Fig. S3). Additionally, high gene coverage was observed from the CF4 patient fingerprick sample after WG-RCA (Supplementary Fig. S3). However, the Comprehensive Cancer Panel is not designed with *ESR1* content, so we opted to use ddPCR for concordance testing instead of targeted sequencing for increased sample throughput, lower cost, and higher detection sensitivity.

### Fingerstick- and venous-based detection of variants is concordant post-whole genome amplification

**Figure 4:**
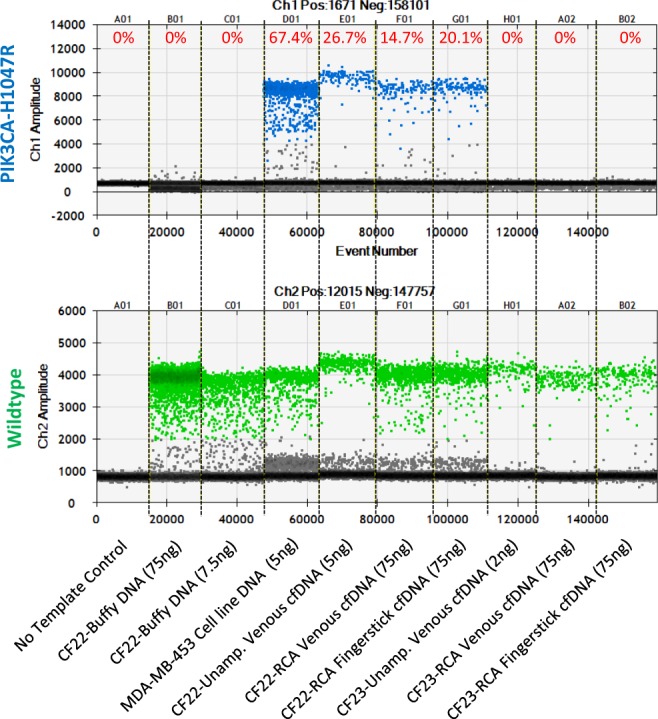
Comparison of PIK3CA-H1047R mutant allele frequencies in patients CF22 and CF23 before and after whole genome rolling-circle amplification (WG-RCA) of venous and fingerstick cfDNA. Genomic DNA from cell line MDA-MB-453 is used as a positive control for the PIK3CA-H1047R mutation. No template control, patient C22 buffy gDNA, and patient CF23 cfDNA samples are used as negative controls for PIK3CA-H1047R. Mutant allele frequencies for patient CF22 are comparable among unamplified cfDNA, WG-RCA venous cfDNA and WG-RCA fingerstick cfDNA. No mutation is detected in patient CF23 samples either before or after WG-RCA.

We utilized droplet digital PCR (ddPCR) to compare sequenced-identified somatic mutations before and after WG-RCA of venous and fingerstick cfDNA (Table 1, Fig. 4, and Supplementary Table S4). Table 1 summarizes the average mutant allele frequencies measured from at least three ddPCR technical replicate runs. Overall, we observed 100% concordance in variant detection before and after WG-RCA of venous cfDNA obtained from patients presenting with circulating mutations (Table 1). Moreover, we observed 100% concordance in variant detection from fingerstick samples processed by WG-RCA compared to unamplified cfDNA obtained by conventional liquid biopsy (Table 1). In some ddPCR tests, mutant allele frequencies showed increased variability after WG-RCA (i.e. non-uniform amplification), possibly due to differential primer access or stochastic amplification of mutant or wildtype alleles. These effects were most evident in patient CF25, who presented with a trace burden (~1.2%) of PIK3CA-E545K by standard liquid biopsy (Table 1). On the contrary, Fig. 4 shows that PIK3CA wildtype and H1047R mutant copy numbers for patient CF22 are comparable before and after WG-RCA of venous and fingerstick cfDNA. Moreover, the H1047R mutation present in a metastatic tumor sample from patient CF23 remained undetectable in all cfDNA samples both before and after WG-RCA of venous and fingerstick cfDNA (Fig. 4). Importantly, quantitative ddPCR results for patients CF23, CF4, CF22, and CF25 (Fig. 4 and Table 1) each compare favorably to allele frequencies obtained by MammaSeq analysis of unamplified venous cfDNA (Supplementary Table S2).

**Table 1:** ddPCR validation of NGS-identified somatic variants in cfDNA before and after whole genome amplification.

Patient ID	NGS-identified variants in cfDNA and/or tumor tissue	Unamplified venous cfDNA	WG-RCA venous cfDNA	WG-RCA fingerstick cfDNA
CF31	PIK3CA-E545K	26.8 ± 2.1	19.7 ± 2.5	22.4 ± 0.7
	KRAS-G12D	23.0 ± 2.2	10.6 ± 0.5	31.8 ± 1.4
CF4	PIK3CA-N345K	16.7 ± 1.0	14.5 ± 7.0	19.0 ± 3.3
	ESR1-D538G	15.0 ± 2.0	2.2 ± 2.0	23.4 ± 7.0
	ESR1-Y537C	10.2 ± 1.6	11.2 ± 1.2	45.9 ± 6.2
CF22	PIK3CA-H1047R	27.9 ± 1.4	15.7 ± 0.6	8.1 ± 3.1
CF25	PIK3CA-E545K	1.2 ± 0.2	11.9 ± 0.8	0.11 ± 0.04

Average mutant allele frequency ±SEM is indicated using data from at least three independent experiments.

While non-uniform amplification is a primary challenge for whole genome amplification techniques, it is important to note that WG-RCA generates very large hyperbranched concatemers that must be fragmented into smaller pieces for efficient droplet partitioning and ddPCR analysis. We down-selected a process that combines both random and non-random fragmentation (Supplementary Fig. S4), namely boiling (Supplementary Fig. S4A) for 5 minutes followed by brief endonuclease treatment during droplet formation, to rapidly process WG-RCA prior to ddPCR. We found that other methods for fragmentation such as g-TUBE centrifugal shear did not work efficiently (Supplementary Fig. S4B), while non-random fragmentation by endonucleases would require significant digestion time (~4hrs) and laborious re-purification to remove salty restriction digest buffers that can inhibit ddPCR according to the BioRad instructions for ddPCR Probe Supermix. Our stream-lined process of using heat and brief endonuclease treatment (without digestion buffer) to fragment WG-RCA prior to ddPCR resulted in reproducible mutant allele detection across a range of template inputs (10–100 ng) for a representative patient (Supplementary Fig. S4C). However, we cannot exclude that random fragmentation and/or inefficient digestion of WG-RCA concatemers might lead to some experimental variability in mutant allele frequency compared to unamplified cfDNA.

### Timeline correlation of CA 27–29 levels with non-concurrent metastatic tumor biopsies and fingerstick blood collection

**Figure 5:**
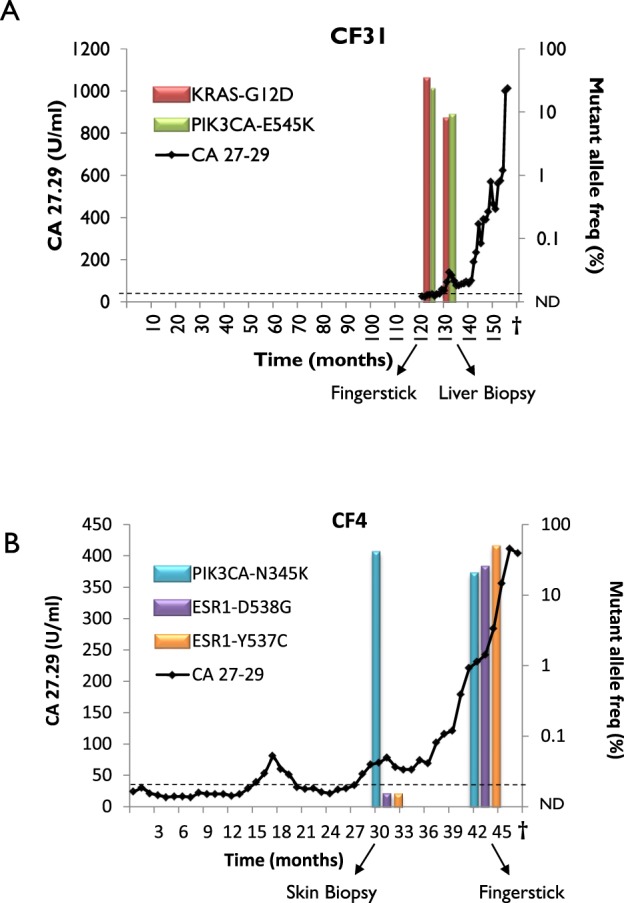
Clinical timelines of CA 27–29, tumor biopsy, and fingerstick blood collection for patients CF31 and CF4. Each timeline starts with diagnosis of metastatic disease. CA 27–29 tumor marker assessments are indicated as line graph. The normal reference range of serum CA 27–29 is less than 38 U/mL. Mutation frequencies are indicated as bar graphs from the ddPCR results in Table 1. The lower limit of detection for ddPCR was set at 0.1% allele frequency.

Serum monitoring of cancer antigen 27–29 levels is routinely used in the clinic to follow treatment response in patients with metastatic breast cancer. To further explore the clinical implications of our pilot findings, we compared the progression of metastatic disease by CA 27–29 in relation to the mutations identified by blood or tumor biopsy (Fig. 5). Retrospective CA 27–29 data were available for patients CF31, CF4, and CF25. For patient CF31, a metastatic liver biopsy conducted 1.5 months after blood collection confirmed the findings of liquid biopsy, namely the presence of *PIK3CA-E545K* and *KRAS-G12D* mutations (Fig. 5A). Patient CF31 displayed minimal or no response to serial treatments of aromatase inhibitor, anti-angiogenic inhibitor, chemotherapy, SERDs, mTOR inhibitor (Everolimus), and cdk 4/6 inhibitor, which correlates with increased CA 27–29 levels over time (Fig. 5A). The lack of response to Everolimus in patient CF31 is consistent with reports that *KRAS* mutations confer resistance to mTOR blockade in the context of *PIK3CA* mutations^25^.

For patient CF4, we previously reported the course of disease while examining the incidence of *ESR1* mutations in advanced breast cancer^15^. Several *ESR1* mutations, namely D538G (5.1%), Y537C (2.7%), and Y537S (1.2%), had been detected by conventional liquid biopsy 6 months after a metastatic skin lesion biopsy, which tested negative for *ESR1* mutations^15^. The patient had received aromatase inhibitor for 6 months prior to detecting circulating *ESR1* mutations, suggesting that these mutations were acquired after the biopsy, and subsequent venous blood draws showed enrichment of D538G (10.1%) and Y537C (7.4%), but loss of the Y537S mutation^15^. In this current study, we re-examined matched fingerstick and venous blood draws that were collected ~12 months after the skin biopsy and confirmed the same mutational profile (Table 1) as reported previously. In addition, an N345K mutation in the *PIK3CA* gene was shared between in the skin biopsy (37.7%) and the fingerstick blood sample (19%). These *ESR1* and *PIK3CA* mutations coincide with increasing CA 27–29 levels in the blood (Fig. 5B), suggesting rapid genomic evolution and tumor progression. Similarly, for patient CF25, a E545K mutation in *PIK3CA* was detected in both fingerstick and venous blood samples (albeit at very low levels) and temporally coincides with high CA 27–29 levels (Supplementary Fig. S5). Collectively, these data suggest that the presence of driver mutations in circulation may indicate the severity of metastatic disease.

## Discussion

The analysis of circulating cell-free DNA (cfDNA) is a promising new strategy for tracking tumor evolution, response to therapy, and predicting relapse often in tandem with medical imaging. Liquid biopsy paradigms are gaining prominence as they are non-invasive, allow real-time monitoring of mutations, and have the potential to capture unbiased tumor heterogeneity. For example, we recently documented the emergence of circulating mutations in estrogen receptor 1 (*ESR1*) among longitudinal blood draws from patients with advanced breast cancer^15,26^, thereby suggesting that tracking these mutations in blood maybe predictive to endocrine resistance. Despite such clinical potential, liquid biopsy analysis of cfDNA has yet to penetrate widely into routine clinical care. A contributing factor may be that robust longitudinal analysis of cfDNA is technically and economically burdensome, given the current requirements for large-volume blood collection, handling, and processing, with exquisite control over pre-analytical variables^11^.

Towards this end, our pilot study provides important physiological evidence that circulating tumor DNA is accessible by fingerstick. We also provide proof-of-concept for collecting and transporting fingerstick samples (centrifuge-free and at ambient temperature) using a paper-based collection device to support whole genome amplification and molecular testing. Several benefits may be realized upon further optimization and refinement of our approach. First, longitudinal cfDNA analysis by fingerstick might be more satisfactory (i.e. less-invasive) for patients because serial venipuncture collection might be regarded as a disincentive. Alternatively, whole genome amplification could augment existing venipuncture practices by reducing the volumetric burden of collecting and processing large volumes of blood to obtain sufficient cfDNA. As illustrated here for patient CF31, whole genome amplification can mitigate instances where archived plasma samples might normally be ignored or discarded due to insufficient cfDNA levels. Finally, others have demonstrated that whole genome amplification increases the sensitivity of detecting mutant alleles in circulation, with important implications for monitoring drug resistance^36^.

By leveraging WG-RCA, we introduce a new approach for liquid biopsy that potentially reduces the need to over-collect patient blood and shows that capillary blood (obtained by fingerprick) is a physiologic access-point for tumor DNA in circulation. By targeted sequence analysis of 409 genes, we demonstrate that WG-RCA improves average depth of coverage and reduces allelic imbalance compared to amplification strategies that are best-suited for long, intact DNA (i.e. multiple displacement amplification, or MDA, for DNA >1 kB). Our findings are consistent with prior cfDNA studies comparing MDA to blunt-end ligation-mediated whole genome amplification (BL-WGA), wherein double-stranded DNA circles were generated by ligation of double-stranded cfDNA prior to rolling-circle amplification^17^. Our WG-RCA method is notably different, in part, because we leverage single-stranded templates of unencumbered flexibility, which increases the efficiency of creating DNA circles by ligation compared to known limitations around the persistence length^37^ and the helical pitch^38^ of double-stranded DNA at sizes below 500 bp. Thus, WG-RCA may efficiently amplify very small cfDNA templates <150 bp that exceed the persistence length limitations of double-stranded DNA and are critical for improving ctDNA detection sensitivity^39^. Nevertheless, with low template input, most whole genome amplification techniques are known to experience stochastic issues, such as allelic drop-out, drop-in, or imbalance, leading to amplification biases or non-uniformity issues that render some methods more suitable than others^40^. Evidence for increased stochasticity in WG-RCA samples is apparent in Table 1, particularly for patient CF25 having a trace burden (~1.2%) of mutant PIK3CA-E545K. However, because rolling circle amplification generates large hyberbranched DNA structures^28^, we cannot exclude that the variability in mutant allele frequency observed in Table 1 might be explained by random fragmentation and/or insufficient digestion of WG-RCA concatemers leading to occluded primer access and poor ddPCR efficiency. As one alternative approach, we previously demonstrated that PCR pre-amplification of a portion of cfDNA generates sufficient template for ddPCR while preserving the linearity of mutant allele detection, albeit at lower throughput for only a few mutations known in advance. This alternative approach might be combined with fingerstick collection in parallel with WG-RCA for monitoring therapy and resistance outcomes.

This current pilot study has certain limitations. To establish proof-of-concept, patient sample size was intentionally limited; follow-on studies are warranted with larger patient cohorts and statistical power to demonstrate a clinical utility for fingerstick detection and/or for individualized monitoring. Further, we did not compare mutation detection in concordant solid tumor biopsy and fingerstick cfDNA, as we feel this is a question best addressed using venous cfDNA. Nonetheless, it is encouraging that our pilot results are consistent with the mutational landscape and ctDNA frequencies reported by a recent liquid biopsy study of 254 patients with metastatic ER-positive breast cancer^12^. While PlasmaClip devices performed suitably for room temperature collection and storage of fingerstick samples within the tested window (6–19 days), further investigations of cfDNA stability as a function of time and temperature are warranted. Lastly, additional optimization of our WG-RCA workflow is necessary to control amplification bias and minimize the possibility of single-stranded DNA circles undergoing spontaneous deamination, which can lead to spurious “jack-pot” C→T transitions^41,42^. One simple solution for controlling amplification bias is to pool two or more individual whole genome amplification products together prior to downstream analysis, as this neutralizes stochastic imbalances which do not re-occur among WGA replicates^43^. Indeed, Shaw *et al*. has reported that pooling triplicate WGA reactions using a commercial MDA kit (illustra^TM^ GenomiPhi^TM^) facilitates accurate liquid biopsy SNP profiling of breast cancer patients^44^. Likewise, spontaneous deamination of single-stranded DNA has been effectively minimized, in other studies, by limiting high-temperature incubations and incorporating DNA repair enzymes prior to rolling circle amplification^42^.

Interestingly, our results show that total cfDNA levels continue to be limiting (i.e. <30 ng/mL of plasma) in advanced disease despite the conventional wisdom that metastatic cancer is easier to detect by liquid biopsy than early disease. Since the conventional wisdom reflects a limit-of-detection for downstream molecular assays (rather than physiological changes in cfDNA), upfront sample collection and processing challenges pertain equally to early and advanced cases for obtaining cfDNA. In this regard, the WG-RCA pre-amplification technique outlined in this study may also benefit early diagnostic detection.

## Methods

### Venous blood and tumor collection

Approximately 40 mLs venous whole blood was drawn in Streck Cell-Free DNA blood tubes from 7 patients (CF4, CF19, CF20, CF22, CF23, CF25, and CF31) with ER-positive metastatic breast cancer seen within the Magee-Womens Hospital of UPMC. All patients had signed informed consent. Venous blood was collected under the University of Pittsburgh IRB approved protocol (IRB0502025) in accordance with IRB guidelines and regulations between June 2014 and February 2015. We have previously reported on the detection of ESR1 mutations in venous plasma cfDNA samples from these 7 patients using ddPCR^15^. MammaSeq testing on venous plasma cfDNA samples from patients CF20, CF22, CF23, and metastatic tumor tissue samples from CF4, CF23, CF31 was described in our previous study^23^. Hence, for 2 patients (CF4 and CF23), MammaSeq^TM^ results from both tumor and blood were available for comparison, albeit at non-concurrent samplings (i.e. blood samples were collected 7 and 12 months after metastatic tumor biopsy, respectively). However, for one patient (CF31), blood was obtained ~1.5 months *prior* to the results of metastatic tumor biopsy but an insufficient plasma volume was banked that yielded less than the required ~20 ng of input cfDNA for MammaSeq analysis. Collection of tumor specimens was described previously^23,45^. CA 27–29 tumor marker levels were obtained from the clinical registry in a de-identified manner.

### Fingerstick blood collection using PlasmaClip

PlasmaClip devices were prototyped by additive manufacturing using a polypropylene-like plastic (Stratasys) on a PolyJet 3D printing system (Stratasys). Glass fiber-based plasma filtration media and cellulose-based plasma collection media (GE Healthcare Life Sciences) were cut into 20 × 8 mm strips and loaded into the PlasmaClip devices to create a precise overlap of 1 mm. Once assembled, PlasmaClip assemblies were sealed in pre-labeled, screw-top specimen jars containing desiccant and shipped to UPMC.

Fingerstick blood was collected (at the same time as venous draws) on the same 7 patients (CF4, CF19, CF20, CF22, CF23, CF25, and CF31) using BD Microtainer Contact-Activated Pink Lancets (BD Diagnostics). All patients had signed informed consent. Fingerstick blood was collected under the University of Pittsburgh IRB approved protocol (IRB0502025) in accordance with IRB guidelines and regulations. Prior to lancing, a pre-selected finger was warmed, wrapped with an elastic rubber band to stimulate engorgement^46^, and then sterilized using an alcohol wipe and fully-dried to avoid spurious hemolysis^47^. After lancing, the first evidence of blood was wiped away and mild but constant pressure was applied to the fingertip to sustain blood follow. A precise volume of 75 μL fingerstick blood was collected using MicroSafe blood collection tubes (Safe-Tec, LLC) and immediately spotted onto PlasmaClip assemblies to fractionate plasma at the point-of-collection. PlasmaClip-based specimens were air-dried for 5–10 minutes to complete the sampling-wicking separation process and then re-sealed in screw-top specimen jars (containing desiccant) for room temperature storage. A single fingerstick specimen was collected per patient for the purposes of this study. Fingerstick blood was also collected from consented healthy donors in accordance with IRB guidelines and regulations (Ethical and Independent Review Services, Study #13095) to develop a methodology for collecting samples without genomic DNA contamination from interstitial fluids.

### DNA extraction

Venous blood drawn into Streck tubes were processed to buffy coat and plasma by centrifugation. Genomic DNA (gDNA) was isolated from buffy coat using DNeasy Blood & Tissue Kit (Qiagen) as per manufacturer’s instructions and eluted in 50 μl of TE buffer. Buffy gDNA was quantified using the Qubit dsDNA BR assay kit (Life Technologies). Matched plasma and PlasmaClip-based samples were shipped to GE Global Research for DNA extraction and whole-genome amplification. Briefly, whole blood specimens collected in Cell-Free DNA Blood Collection Tubes (Streck, Inc.) were initially centrifuged at 1600xg (10 minutes, 4 °C) to isolate plasma and buffy coat, followed by a secondary clarification spin at 1600xg (10 minutes, 4 °C) and a final high-speed spin at 16,000xg (10 minutes, 4 °C) to isolate cell-free plasma, which was subsequently frozen at −80 °C until use. Dried PlasmaClip-based specimens were stored at room temperature in a desiccator cabinet until use.

To extract venous cfDNA, frozen plasma samples were thawed, and 1–2 mL volumes of cell-free plasma were extracted using the QIAamp^TM^ Circulating Nucleic Acid kit (Qiagen) with a QIAVac 24 manifold. Manufacturer instructions were followed with exception that carrier RNA was omitted from Buffer ACL as this interfered with downstream cfDNA analysis. Column-captured cfDNA was eluted using 22 μL of Buffer AVE. cfDNA was quantified using Quant-iT™ PicoGreen® dsDNA Kit (ThermoFisher Scientific)

To extract fingerstick cfDNA, plasma collection strips were removed from archived PlasmaClip-based specimens and extracted using the DNA Extractor SP kit (Wako Pure Chemical Industries, Ltd.) with minor modification to the manufacturer protocol. Pelleted cfDNA was washed with 100% ACS-grade ethanol prior to rehydration in a minimal volume (~5 μL) of 10 mM HEPES (pH 8.0), 0.1 mM EDTA, and 0.01% Tween-20.

### Whole genome rolling-circle amplification

Approximately 15% of purified venous cfDNA (3 μL) and 100% of purified fingerstick cfDNA (5 μL) were amplified by whole genome rolling-circle amplification. Briefly, cfDNA was heated to separate strands, ligated to form single-stranded DNA circles, and amplified using phi29 polymerase and exonuclease-resistant random primers. All steps were performed in a single-tube format using a proprietary workflow that eliminates the requirement for intermediate sample clean-up and therefore avoids cfDNA template loss during processing. WG-RCA reaction components (i.e. phi29 enzyme, primer, and buffer) were pre-cleaned of possible DNA contaminants using the exonuclease activity of phi29 prior to the addition of template and dNTPs. Following whole genome amplification, the amplified cfDNA product was purified using SureClean Plus (Bioline Reagents) and resuspended in 10 mM HEPES (pH 8.0), 0.1 mM EDTA, and 0.01% Tween-20. DNA concentrations of purified WG-RCA DNA were quantified using the Quant-iT dsDNA Broad-Range Assay Kit (ThermoFisher Scientific).

### Targeted sequencing

20 ng of input DNA was used for target amplification and library preparation using Ion AmpliSeq™ Library Kit 2.0 (Life Technologies) and the MammaSeq^TM^ panel as described previously^23^. Template preparation by emulsion PCR and enrichment was performed on the Ion OneTouch 2 system (Life Technologies). Template positive Ion Sphere particles (ISP) were loaded onto P1 chip (cfDNA, 5000×) and 318 chips (buffy coat DNA, 500×), followed by sequencing on the Ion Proton^TM^ (Life Technologies) and Ion Torrent Personal Genome Machine (PGM^TM^) (Life Technologies) respectively. Mean read depth is indicated for all cfDNA and buffy gDNA samples in Supplementary Fig. S6.

### Variant Calling

Sequencing data was analyzed using Torrent Suite V4.0 software and Variant Caller plug-in. Variants were evaluated using Ingenuity Variant Analysis (IVA) tool. Mutant allele frequency cut-off was set to 1%. Systematic filtering steps were incorporated to accurately identify true somatic variants. Germline variants were removed from matched cfDNA-buffy gDNA pairs within individual patient samples. CRAVAT 4 (Cancer-Related Analysis of Variants Toolkit) was used to annotate remaining variants. Further, variants occurring in 1000Genomes were removed. Additional filtering was performed to eliminate errors due to strand-bias and preserve the protein-coding and non-synonymous variants. Finally, variants were visually inspected using Integrative Genomics Viewer (IGV) software.

### ddPCR

For all ddPCR runs, matched cfDNA and genomic DNA (g-DNA) derived from buffy coat were included to confirm that the mutation was somatic. Purified WG-RCA DNA was heated at 95 °C for 5 min and then cooled on ice prior to analysis. Approximately 75 ng of WG-RCA DNA, 7.5–75 ng of buffy gDNA, and 1–14 ng of unamplified cfDNA were used per ddPCR reaction. For buffy gDNA and WG-RCA DNA, restriction enzyme was included during droplet generation using the QX100 Droplet Digital PCR System (Bio-Rad Laboratories). No template control and g-Blocks bearing mutation of interest or DNA from a cell line with mutation as positive controls were included in each run. Allele frequency of 0.1% was used as a lower limit of detection based on background mutant signal detected in some buffy gDNA samples. The following PrimePCR ddPCR assays (Bio-Rad Laboratories) were analyzed: dHsaCP2000078 (PIK3CA)/dHsaCP2000077 (H1047R), dHsaCP2000076 (PIK3CA)/dHsaCP2000075(E545K), and dHsaCP2000002 (KRAS)/dHsaCP2000001(G12D). Custom ddPCR assays were developed for PIK3CA-N345K (Life Technologies), ESR1-D538G (Integrated DNA Technologies), and ESR1-Y537C (Life Technologies) as described in Supplementary Table S5.

## Electronic supplementary material

Supplementary data

Supplementary Table S1

Supplementary Table S4

## Data Availability

ddPCR data analyzed during this study are included in this published article and additional information files. MammaSeq datasets analyzed during the current study are not publicly available but can be obtained from the corresponding author on request following appropriate institutional approvals.
