# Supplementary material for: Whole genome amplification of cell-free DNA enables detection of circulating tumor DNA mutations from fingerstick capillary blood
Source: Sci Rep. 2018 Nov 23;8:17313. doi: 10.1038/s41598-018-35470-9 (PMC6251935; doi:10.1038/s41598-018-35470-9)
Supplement: Supplementary file 1 — Supplementary data [file 41598_2018_35470_MOESM1_ESM.pdf]

Whole genome amplification of cell-free DNA enables detection of circulating tumor DNA mutations from fingerstick capillary blood

Rekha Gyanchandani,[1†] Erik Kvam,[2*] Ryan Heller,[2‡] Erin Finehout,[2††] Nicholas Smith,[1] Karthik Kota,[3] John R. Nelson,[2] Weston Griffin,[2] Shannon Puhalla[1], Adam M. Brufsky[1], Nancy E. Davidson[1], and Adrian V. Lee[1*]

[1] Women's Cancer Research Center, Department of Pharmacology and Chemical Biology, University of Pittsburgh Cancer Institute, Magee Womens Research Institute, Pittsburgh, PA, 15213, USA

[2] GE Global Research, One Research Circle, Niskayuna, NY, 12309, USA

[3] Department of Medicine, University of Pittsburgh, Pittsburgh, PA, 15213, USA

[†] Current address: Western Oncolytics, 265 William Pitt Way, Pittsburgh, PA, 15238, USA

[‡] Current address: QIAGEN, 100 Cummings Center, Beverly, MA, 01915, USA

[††] Current address: Terumo BCT, 10811 Collins Ave, Lakewood, CO, 80215, USA

*Corresponding authors:

Adrian V. Lee, Ph.D.
Phone: (412) 641-8554
Fax: (412) 641-2458
Mail: Magee Womens Research Institute, 204 Craft Avenue, Room A412, Pittsburgh, PA, 15213
Email: leeav@upmc.edu

Erik Kvam, Ph.D.
Phone: (518) 387-4289
Fax: (518) 387-7765
Mail: GE Global Research, One Research Circle, K1 5D29, Niskayuna, NY, 12309
Email: kvame@ge.com

Short Title: Cell-free DNA amplification from fingerstick blood

Keywords: Fingerstick, venous blood, cell-free DNA, rolling circle amplification, metastatic breast cancer, somatic mutations, liquid biopsy

Supplementary Methods

Optimization of fingerprick blood collection for low gDNA contamination

Fingerprick blood was collected from healthy donors under IRB (Ethical and Independent Review Services, Study #13095). Procedural parameters were systematically analyzed, including (1) type of lancet, (2) effect of milking, (3) finger selection, (4) tourniquet options, and (5) effect of residual ethanol on the finger after surface decontamination. Two types of commercial lancets were tested, namely a pressure-activated lancet (BD Microtainer Pink, BD Diagnostics) and a spring-activated lancet (UniStik 3 Dual, Owen Mumford). Prior to lancing, the pre-selected finger was warmed and then sterilized with an alcohol wipe. After lancing, the first evidence of blood was wiped away and then pressure was applied to the finger to sustain blood flow. In further modifications, the finger was wrapped with an elastic rubber band to stimulate engorgement prior to lancing, and any residual alcohol on the fingertip was fully-dried to avoid spurious hemolysis. A precise volume of 75μL capillary blood was collected using MicroSafe blood collection tubes (Safe-Tec, LLC) and immediately spotted onto PlasmaClip to fractionate plasma at the point-of-collection. PlasmaClip specimens were air-dried for 5-10 minutes to complete the sampling-wicking separation process and subsequently stored at room temperature in a desiccator cabinet for at least 3 days. To extract capillary cfDNA, plasma collection strips were removed from the PlasmaClip specimens and extracted using the DNA Extractor SP kit (Wako Pure Chemical Industries, Ltd.) with minor modification to the manufacturer protocol. Pelleted DNA was washed with 100% ACS-grade ethanol prior to rehydration in 10μL of 10 mM HEPES (pH 8.0), 0.1 mM EDTA, and 0.01% Tween-20. DNA samples were electrophoretically separated in a 2% agarose gel using Tris/borate/EDTA buffer

solution and stained with SYBR Gold (Thermo Fisher Scientific) for ultrasensitive detection using a Typhoon Imager (GE Healthcare).

Targeted sequencing comparison of healthy donor cfDNA before and after whole genome amplification

Venous whole blood from a pregnant donor was purchased from BioreclamationIVT and collected into Cell-free DNA Blood Collection Tubes (Streck, Inc). Cell-free plasma was isolated by differential centrifugation (1600xg, 10 minutes, 4°C to isolate plasma, followed by a secondary clarification spin at 1600xg, 10 minutes, 4°C; and a final high-speed spin at 16,000xg, 10 minutes, 4°C to isolate cell-free plasma). cfDNA was purified from 200µL aliquots of plasma using the DNA Extractor SP kit (Wako Pure Chemical Industries, Ltd) and pooled. The donor was found to contain ~52ng of cfDNA in 0.8mL of plasma, consistent with the elevated concentration of cfDNA during pregnancy. Approximately 1ng of purified cfDNA was either circularized or maintained as linear DNA and then amplified by rolling circle amplification (WG-RCA) or multiple-strand displacement (MDA), respectively. For WG-RCA, cfDNA was first heated to separate strands, ligated to form single-stranded DNA circles, and amplified using phi29 polymerase and exonuclease-resistant random primers. All steps were performed in a single-tube format using a proprietary workflow that eliminates the requirement for intermediate sample clean-up and therefore avoids cfDNA template loss during processing. All WG-RCA and MDA reaction components (i.e. phi29 enzyme, primer, and buffer) were pre-cleaned of potential DNA contaminants—using the exonuclease activity of phi29—prior to the addition of template and dNTPs. Amplified and unamplified cfDNA samples were submitted to GeneDx for next-generation sequencing using the Ion AmpliSeq Comprehensive Cancer

Panel (Thermo Fisher Scientific), which comprised 409 target genes at all-exon coverage. Sequencing results were compared by plotting the average depth of coverage per target gene as a function of chromosomal location.

Comparison of cfDNA sequence depth before and after whole genome amplification using blood from patient CF4

Matched venous and fingerprick blood samples were collected from patient CF4 and cfDNA was isolated and amplified as described in the Methods section. For venous blood, we obtained 304ng of cfDNA from 2 ml of plasma from this atypical case, and column-purified cfDNA was eluted at a final concentration of 13.8 ng/µL. Approximately 41.4 ng (3 µL) and 34.5ng (2.5 µL) of purified venous cfDNA was used as template for WG-RCA or MDA, respectively. WG-RCA was performed as described in the Methods section for fingerstick and venous cfDNA. MDA was carried out using REPLI-g Mini Kit (Qiagen) according to manufacturer instructions. Amplified and unamplified cfDNA samples were submitted to Genomics Research Core (GRC) at the University of Pittsburgh for next-generation sequencing using the Ion AmpliSeq Comprehensive Cancer Panel (Thermo Fisher Scientific), followed by average coverage analysis per gene.

Comparison of WG-RCA fragmentation methods for ddPCR mutation detection using patient CF31 venous cfDNA

WG-RCA comprising highly-branched, concatemeric DNA was evaluated by gel electrophoresis after fragmentation via one or more of the following methods: centrifugal-based shearing using g-TUBE™ (Covaris, Inc.) according to the manufacturer 6kB protocol, 4-hour restriction digest using MseI endonuclease in 1x CutSmart® buffer (NEB)

at 37°C, or boiling in HET buffer (10 mM HEPES pH 8.0, 0.1 mM EDTA, 0.01% Tween-20) at 95°C. Treated WG-RCA samples were then analyzed by ~1% agarose gel electrophoresis (either native or alkaline) with SYBR Gold (Thermo Fisher Scientific) to assess DNA fragmentation patterns. For mutation detection by ddPCR, WG-RCA was either heated at 95°C for 5 minutes or subjected to g-TUBE centrifugal shear prior to short incubation with MseI endonuclease (but not CutSmart buffer) during droplet generation on the QX100 Droplet Digital PCR System (Bio-Rad Laboratories). MseI is recommended for ddPCR SuperMix for Probes (Bio-Rad) according to the manufacturer without restriction digest buffer. Approximately 10ng, 75ng, and 100ng of WG-RCA was fragmented prior to ddPCR analysis, and mutation detection (PIK3CA-E545K) was compared against unamplified venous cfDNA (1-3ng). Based on these ddPCR results, 75ng of WG-RCA was down-selected for optimal wildtype allele content to ensure robust Poisson-detection of rare mutation events. Heat-based fragmentation (5 min, 95°C) in combination with endonuclease treatment during droplet formation was down-selected for ease of use with multiple WG-RCA samples.

Supplementary Figure S1

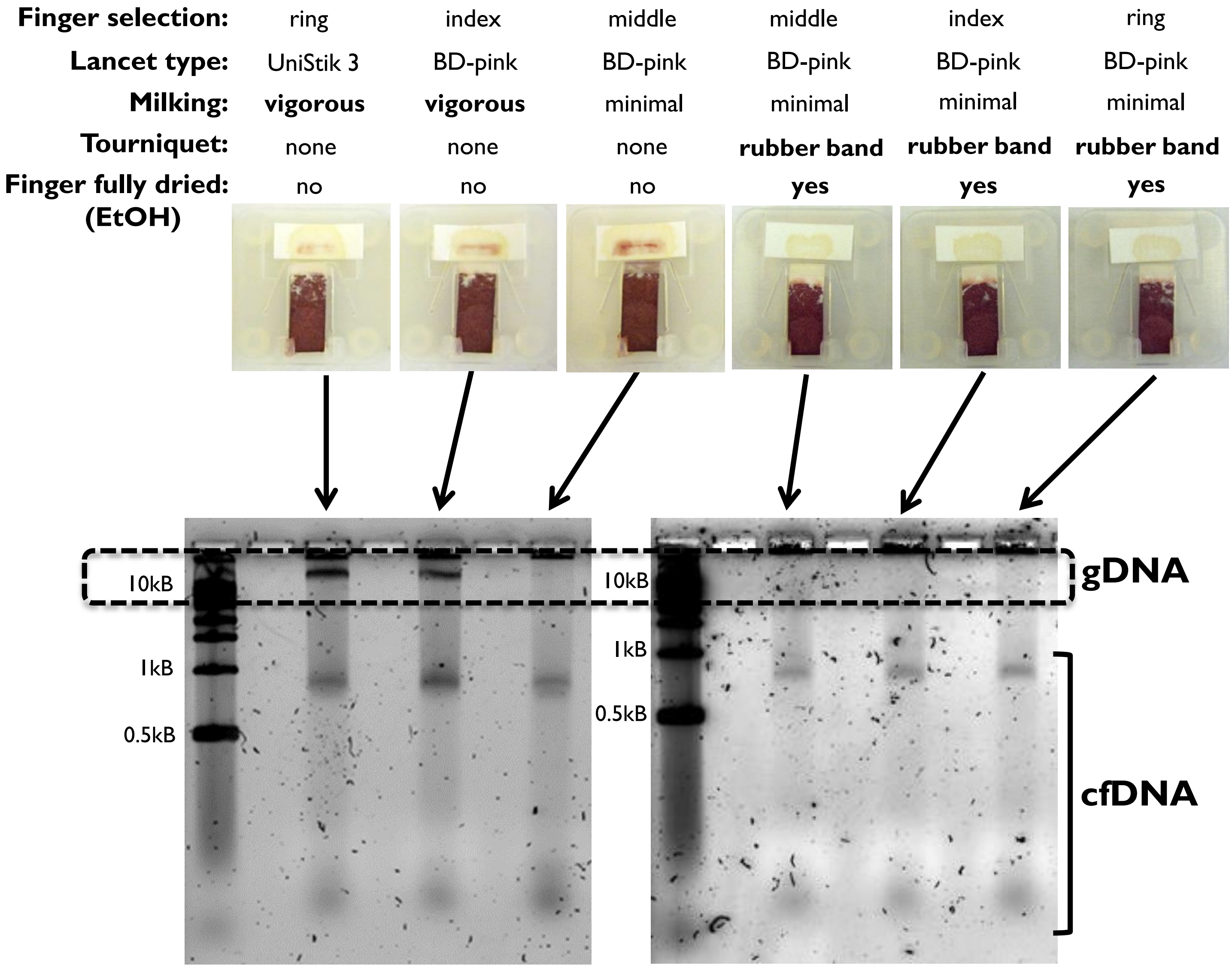

Figure S1: Fingerstick procedure optimization using standard lancing equipment on healthy donors. Fingerstick collection was optimized to avoid contamination of plasma with genomic DNA from interstitial fluids released during vigorous massaging of the finger.

Supplementary Figure S2

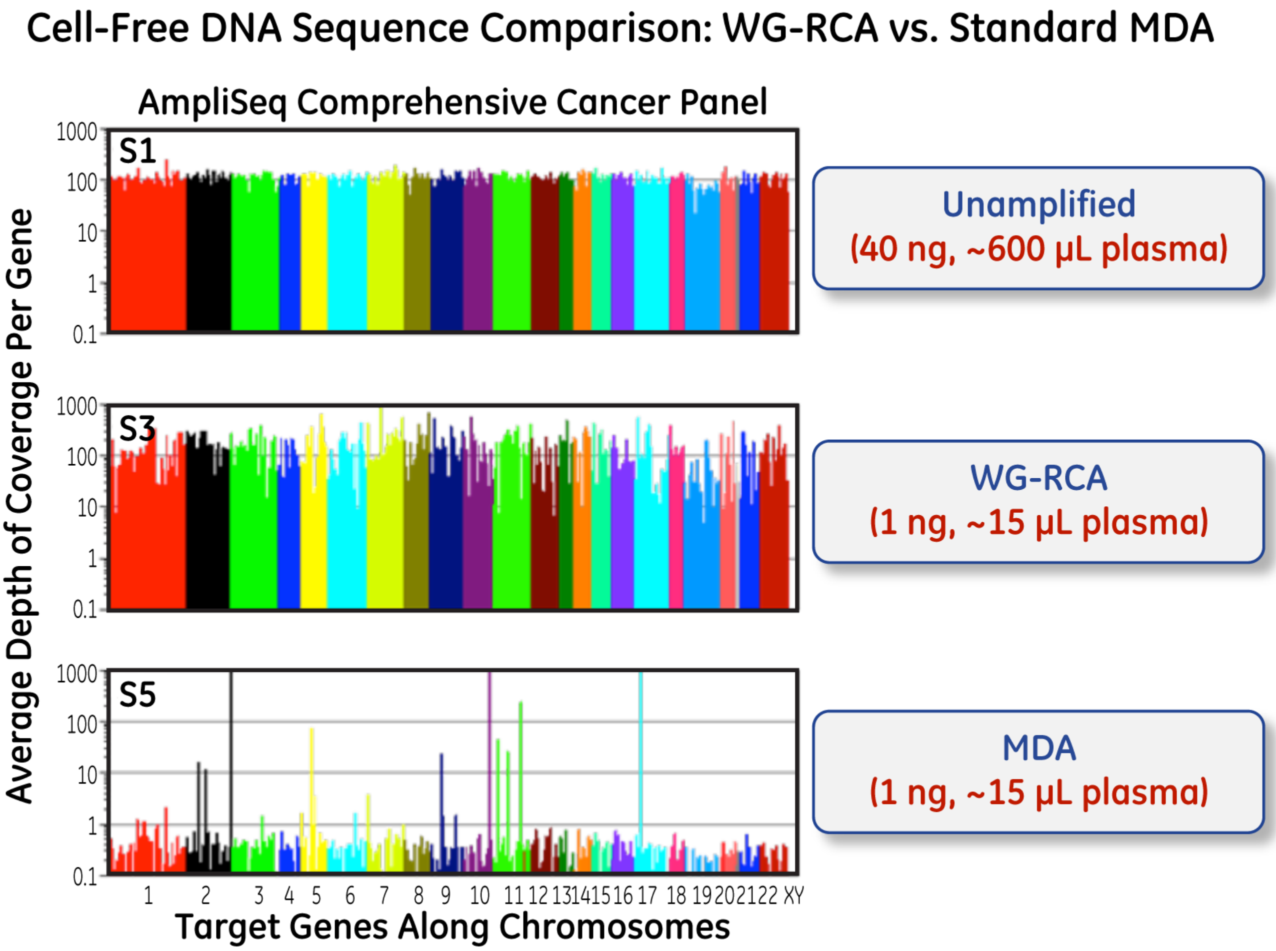

Figure S2: Comparison of whole genome amplification methodology to NGS target coverage using cfDNA from a healthy donor. Whole-genome rolling circle amplification (WG-RCA) of a small amount of circularized single-stranded cfDNA (~1ng, corresponding to ~15uL of donor plasma) increases NGS coverage for a 409-gene target panel compared to multiple displacement amplification (MDA) of linear cfDNA. Phi29 DNA polymerase was utilized in both methods.

Supplementary Figure S3

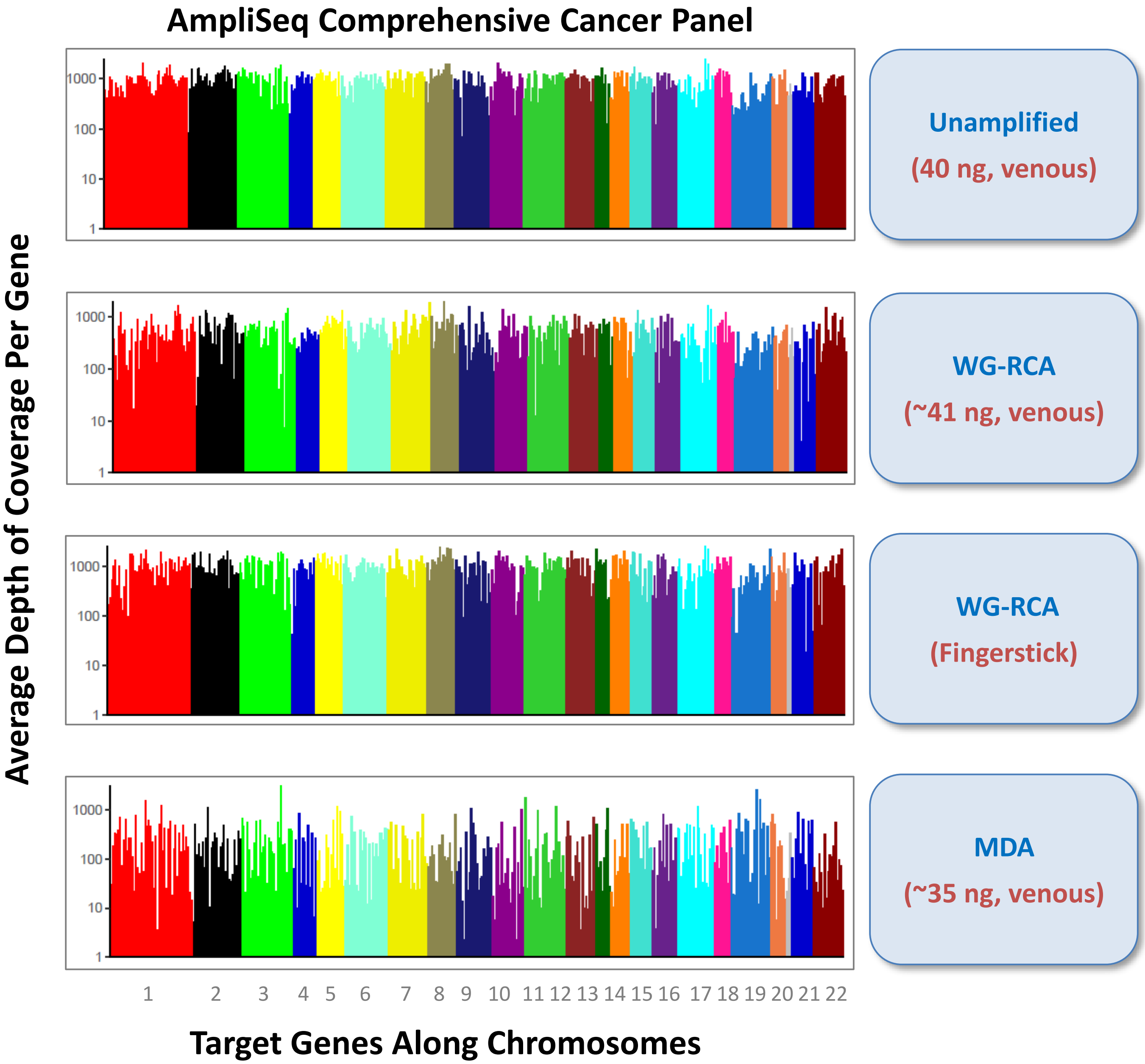

Figure S3: Comparison of whole genome amplification methodology to NGS coverage using cfDNA from patient CF4. The patient fingerstick sample and 35-40ng venous cfDNA template were subjected to whole genome amplification using WG-RCA or MDA, followed by targeted sequencing using Ion AmpliSeq Comprehensive Cancer Panel. Average depth of coverage per gene is indicated for unamplified cfDNA, venous WG-RCA, fingerstick WG-RCA, and venous MDA samples. Both venous- and fingerstick WG-RCA show improved coverage relative to venous MDA.

Supplementary Figure S4

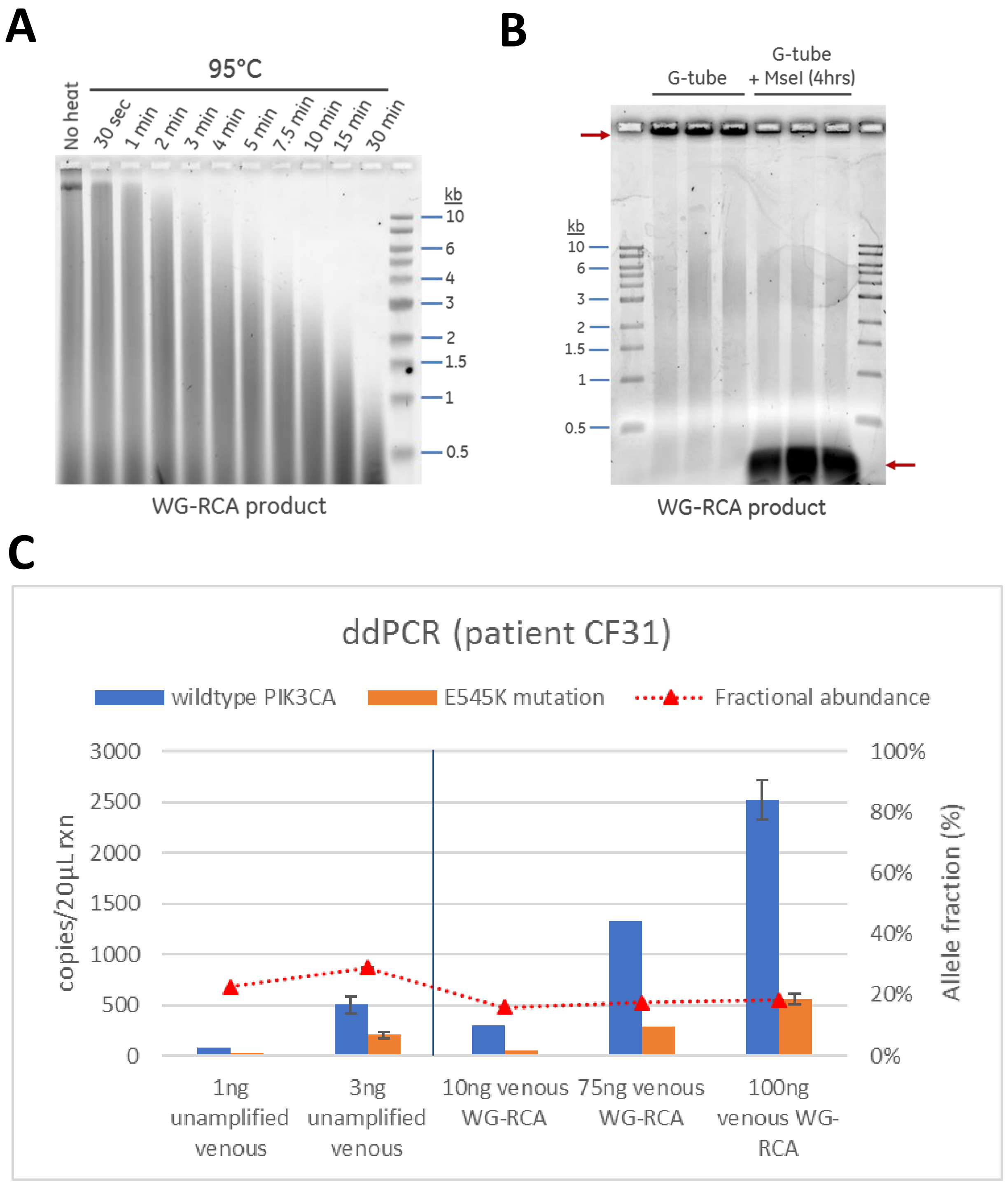

Figure S4: Fragmentation methods to optimize WG-RCA template prior to ddPCR. A) Purified WG-RCA DNA was heated at 95 °C for different durations (ranging 30sec – 30min) and then cooled on ice prior to gel electrophoresis analysis. **B)** Fragmentation of WG-RCA using g-TUBE centrifugal shear with and without restriction endonuclease. **C)** PIK3CA-E545K mutant allele detection across a range of WG-RCA template inputs (10 -100 ng) following fragmentation using heat (95 °C for 5min) and brief endonuclease treatment (without digestion buffer).

Supplementary Figure S5

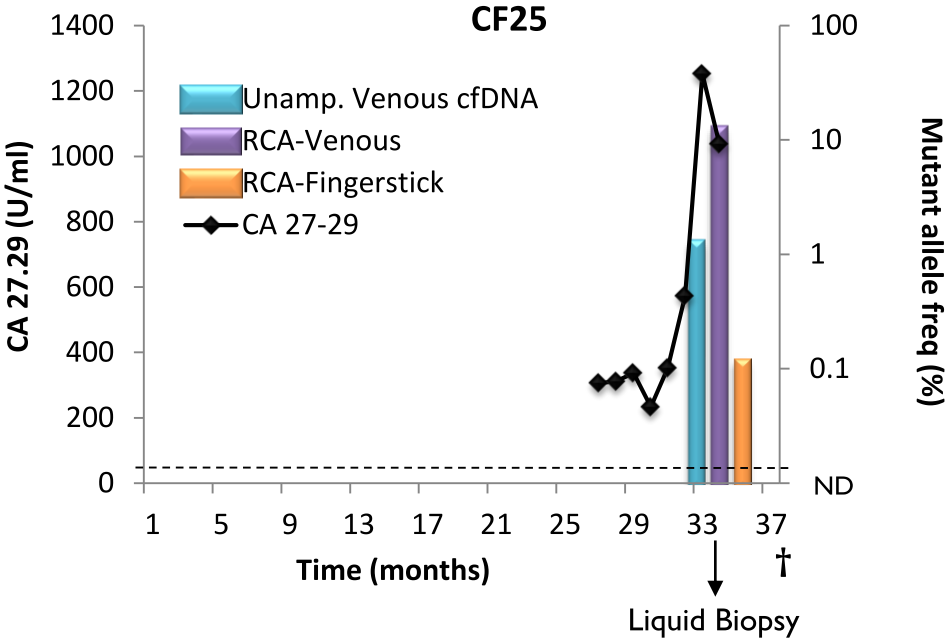

Figure S5: Clinical timeline and PIK3CA-E545K mutation detected in venous and fingerstick blood for patient CF25. The timeline starts with diagnosis of metastatic disease. CA 27-29 tumor marker assessments are indicated as line graph. The normal reference range of serum CA 27-29 is less than 38 U/mL. Mutation frequencies are indicated as bar graphs. The lower limit of detection for ddPCR was set at 0.1% allele frequency.

Supplementary Figure S6

A

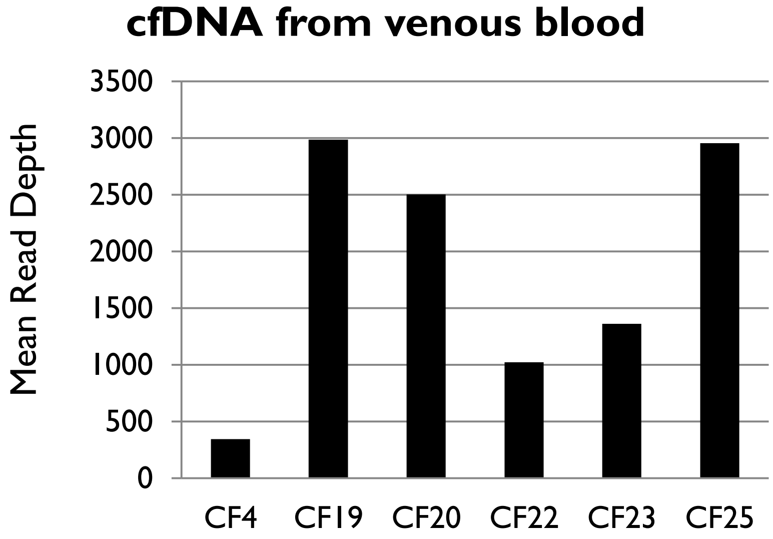

B

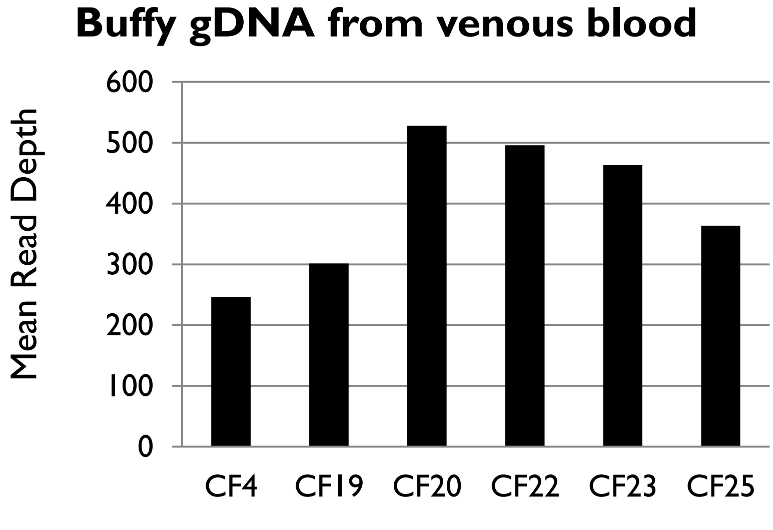

Figure S6: Quality metrics for Ion Torrent AmpliSeq. P1 chip (cfDNA, 5000x) and 318 chip (buffy g-DNA, 500x) were used to run the MammaSeq^{TM} panel on the Ion Proton^{TM} (Life Technologies) and Ion Torrent Personal Genome Machine (PGM^{TM}) (Life Technologies) respectively. Mean read depth is indicated for all cfDNA **(A)** and buffy g-DNA **(B)** samples.

Supplementary Table S2

Patient ID	Venous cfDNA	Buffy Coat gDNA	Metastatic Tumor DNA	Time Between Collection of Tumor and Blood Sample
CF31	Insufficient amount	Not sequenced	PIK3CA-E545K (8.7%)<br>KRAS-G12D (7.4%)	(-)1.5 months
CF4	PIK3CA-N345K (10.0%)<br>ESR1-D538G (8.3%)<br>ESR1-Y537C (8.4%)	Not detected	PIK3CA-N345K (37.7%)<br>Not detected<br>Not detected	(+)12 months
CF19	Not detected	Not detected	No tumor collected	No tumor collected
CF20	Not detected	Not detected	No tumor collected	No tumor collected
CF22	PIK3CA-H1047R (32.0%)	Not detected	No tumor collected	No tumor collected
CF23	Not detected	Not detected	PIK3CA-H1047R (27.9%)	(+)7 months
CF25	PIK3CA-E545K (1.1%)	Not detected	No tumor collected	No tumor collected

Supplementary Table S2: NGS-identified variants and mutant allele frequencies (%) for patient-matched cfDNA, buffy gDNA, and metastatic breast tumor samples. Time between collection of tumor tissue and blood is indicated as minus (-) if blood was collected prior to the tumor sample and vice versa. All samples were sequenced using MammaSeq, which covers 79 genes frequently mutated in breast cancer.

Supplementary Table S3

Patient ID	Fingerstick Collection Date	Shipment Arrival at GEGR	cfDNA Extraction	Days ambient (total)
CF31	24-Jun	1-Jul	2-Jul	8 days
CF4	26-Jun	1-Jul	2-Jul	6 days
CF19	15-Jul	18-Jul	23-Jul	8 days
CF20	24-Jul	29-Jul	12-Aug	19 days
CF22	5-Aug	11-Aug	12-Aug	7 days
CF23	7-Aug	12-Aug	13-Aug	6 days
CF25	14-Aug	19-Aug	26-Aug	12 days

Table S3: Storage durations of PlasmaClip fingerstick samples from the time of collection to cfDNA extraction. A single dried plasma spot was obtained from each of the 7 breast cancer patients and stored at room temperature for a total of 6-19 days.

Supplementary Table S5

Mutation	Forward primer	Reverse primer	Mutant Probe	WT probe	Fluorescence
PIK3CA-N345K	CCCTTTGGGTTATAAATAGTGCACTCA	AGCATCAGCATTTGACTTTACCTTATCA	CCTACGTGAAAGTAAATA	AACCTACGTGAATGTAAATA	FAM/VIC
ESR1-D538G	GCATGAAGTGCAAGAACGTG	AAGTGGCTTTGGTCCGTCT	TCTATGGCCTGCTGCTGGAGATGCT	TCTATGACCTGCTGCTGGAGATGCT	HEX/FAM
ESR1-Y537C	CAGCATGAAGTGCAAGAACGT	TGGGCGTCCAGCATCTC	CCCCTCTGTGACCTG	TGCCCCTCTATGACCTG	FAM/VIC

Table S5: Sequence of ddPCR primers and probes. Custom ddPCR assays were developed for PIK3CA-N345K (Life Technologies), ESR1-D538G (Integrated DNA Technologies), and ESR1-Y537C (Life Technologies).
